# Pertussis outbreak in university students and evaluation of acellular pertussis vaccine effectiveness in Japan

**DOI:** 10.1186/s12879-015-0777-3

**Published:** 2015-02-06

**Authors:** Megumi Hara, Mami Fukuoka, Katsuya Tashiro, Iwata Ozaki, Satoko Ohfuji, Kenji Okada, Takashi Nakano, Wakaba Fukushima, Yoshio Hirota

**Affiliations:** Department of Preventive Medicine, Faculty of Medicine, Saga University, 5-1-1 Nabeshima, Saga City, Saga 849-8501 Japan; Department of Infection Control, Saga-ken Medical Centre Koseikan, 400 Nakahara, Kase, Saga City, Saga 840-8571 Japan; Department of Pediatrics, Faculty of Medicine, Saga University, 5-1-1 Nabeshima, Saga City, Saga 849-8501 Japan; Health Care Center, Saga University, 5-1-1 Nabeshima, Saga City, Saga 849-8501 Japan; Department of Public Health, Faculty of Medicine, Osaka City University, 1-4-3, Asahi-machi, Abeno-ku, Osaka 545-8585 Japan; Department of Pediatrics, Fukuoka Dental College, 2-15-1 Tamura, Sawara-ku, Fukuoka City, Fukuoka 814-0193 Japan; Department of Pediatrics, Kawasaki Medical School, 577 Matsushima, Kurashiki City, Okayama 701-0192 Japan; Clinical Epidemiology Research Center, Medical Co. LTA, 6-18, Ten-ya-machi, Hakata-ku, Fukuoka, 812-0025 Japan

**Keywords:** Pertussis, Outbreak, Vaccine effectiveness

## Abstract

**Background:**

Recent studies worldwide have reported increasing numbers of adults diagnosed with *Bordetella pertussis* despite receiving childhood vaccinations. This study describes a pertussis outbreak at a university medical faculty campus and examines the effectiveness of diphtheria, tetanus, and pertussis (DTaP) vaccination completed during infancy in Japan.

**Methods:**

After the outbreak, self-administered questionnaires and serum samples were collected from students on campus to determine the incidence of pertussis and underlying diseases. Pertussis was diagnosed on the basis of clinical criteria and serum anti-pertussis toxin antibody levels. Using data collected from 248 first and second grade students who had submitted copies of their vaccination records, we evaluated the effectiveness of DTaP vaccination in infancy against adult pertussis.

**Results:**

Questionnaire responses were obtained from 636 students (of 671 registered students; 95% response rate). Of 245 students who reported a continuous cough during the outbreak period, 84 (attack rate: 13.2%) were considered “probable” pertussis cases that met clinical criteria. The outbreak occurred mainly in first and second grade students in the Faculty of Medicine. Of 248 students who provided vaccination records, 225 had received 4 DTaP doses (coverage: 90.7%); the relative risk of the complete vaccination series compared to those with fewer than 4 doses or no doses for probable cases was 0.48 (95% confidence interval: 0.24-0.97).

**Conclusions:**

Waning protection was suspected due to over time. Booster vaccination for teenagers and development of highly efficacious pertussis vaccines are needed.

**Electronic supplementary material:**

The online version of this article (doi:10.1186/s12879-015-0777-3) contains supplementary material, which is available to authorized users.

## Background

Although global vaccination coverage for diphtheria, tetanus, and pertussis (DTaP) remains high, recent reports of increasing pertussis cases among adolescents and adults are of concern because this population can be a source of infant infection [1]. Suggested causes for this increase include increased clinical awareness of pertussis, improved diagnostics using polymerase chain reaction (PCR), identification of mutations in the strain of *Bordetella pertussis* associated with epidemics, and decreasing antibody titers after vaccination [2-6]. Western countries have initiated tetanus, reduced-antigen-content diphtheria, and acellular pertussis (Tdap) vaccine booster programs for adolescents, adults, and other high-risk groups [1,7,8].

The number of adult pertussis cases has been increasing in Japan, with outbreaks in high schools and universities as well as workplaces successively reported in 2002 [9-13]. In response to these reports, studies have examined outbreak characteristics, genetic characteristics of *B. pertussis*, and alternative diagnostic methods. However, to our knowledge, no study has evaluated the effectiveness of the current DTaP vaccine. Japan has a different schedule to western countries for baby immunizations, including DTaP vaccine. Until 2012, pertussis vaccination is a triple DTaP vaccine (after 2012, DTaP-IPV), beginning at 3 months of age. To establish initial immunity, 3 times for 3 to 8 weeks apart are needed. A booster dose is given at 6 months to 12 months after the initial immunity. Thus, DTaP vaccine is usually completed by 18 months of age. The recommended number of doses is smaller than that in Western countries. In addition, Tdap booster vaccines are not administered after early adolescence in Japan. To determine the necessity for booster vaccination in early adolescence, it is important to evaluate the effectiveness of the current vaccine program in preventing pertussis after early adolescence. However, there have been a limited number of epidemiological evaluations on vaccine program effectiveness against pertussis in Japan, and these studies have focused primarily on children [14,15]. To our knowledge, no studies have examined the effectiveness of the vaccine against pertussis after early adolescence.

In April 2010, a pertussis outbreak was confirmed among students at the medical faculty campus of Saga University. After the outbreak ended, a retrospective cohort study was performed. This study describes the outbreak and examines the association between infant DTaP vaccination and incidence of pertussis.

## Methods

### Study populations

More than 20 students visited the health administration center at the Saga University Faculty of Medicine in April 2010 complaining of coughs that had lasted at least 2 weeks. Three of these students had throat swabs positive for *B. pertussis* by loop-mediated isothermal amplification [16]. Thus, this outbreak of cough symptoms was considered to be due to pertussis. The health administration center discouraged club activities, meetings, and ball game tournaments; promoted use of facemasks; terminated practical training for students with coughs; actively encouraged medical examinations at medical institutions; and notified students and faculty members of the outbreak by e-mail. By early July, no new cough cases were reported to the health administration center.

Just after the end of the outbreak in early July, a total of 671 students (411 and 260 from the departments of medicine and nursing, respectively) from the first through fourth grades on the faculty of medicine campus were provided an oral explanation of the purpose, content, and conditions of cooperation of the study, and asked to provide written informed consent forms with agreement to participate. Among them, 636 students (collection rate: 95%) completed a questionnaire about relevant demographic variables and clinical symptoms of cough, including duration, presence of coughing paroxysms, whooping and vomiting after cough, medical institution visits, past history of disease, and DTaP vaccination status. They were also asked to provide serum specimens. Serum samples were obtained from 516 (77.1%) of these students; anti-pertussis toxin (PT) antibody levels were tested by enzyme immunoassay at an outside laboratory (SRL, Inc., Tokyo).

Of these, 248 first and second grade students had submitted copies of their vaccination records, including infant DTaP vaccine administration histories, from their maternity record books to the health administration center upon entering the school. In Japan, vaccination histories are recorded in maternity record books maintained by individuals.

This study design was approved by the ethical review board of the Saga Medical School Faculty of Medicine, Saga University (approval number 22–25, 2010).

### Case definitions

Cases were categorized on the basis of 2 clinical definitions of pertussis, using clinical criteria established by the Centers for Disease Control and Prevention and the Council of State and Territorial Epidemiologists 2014 case definitions [17]. “Probable cases” had cough illness lasting ≥2 weeks with at least 1 of the following signs or symptoms: paroxysms of coughing, or inspiratory “whoop”, or posttussive vomiting. “Suspected cases” met at least 1 of the 4 clinical symptoms or signs. In addition to these clinical definitions, the serological diagnosis of pertussis required serum anti-PT antibody levels after the outbreak to be higher than 100 EU/mL.

### Vaccine effectiveness

The 248 students whose vaccination records could be confirmed by their maternity record books were classified into 2 groups: those who had completed the full 4-dose vaccination as recommended by the Japanese government, those who had received less than 4 vaccine doses or no doses. The attack rate (AR) of pertussis and the relative risk (RR) after 4 doses compared with less than 4 doses or no doses were calculated. The effectiveness of the vaccine was calculated using the equation:

$$ \left(1 - \left[A{R}_{vaccinated}/A{R}_{unvaccinated}\right]\right) \times 100\ \left(\%\right) = \left(1\ \hbox{--}\ RR\right) \times 100\ \left(\%\right) $$

### Statistical analysis

We used SAS 9.3 for Windows (SAS Institute, Cary, NC, USA) for statistical comparisons between each variable using chi-square and Fisher’s exact tests. RRs after 4 doses compared with less than 4 doses or no doses and corresponding 95% confidence intervals (CIs) were obtained using the PROC FREQ procedure in the software package. RRs and their 95% CIs adjusted by faculty were obtained using the Mantel–Haenszel method.

## Results

### Description of outbreak

The population characteristics and cough statuses of 636 subjects who participated in the survey just after the outbreak were examined according to clinical diagnosis (Table 1). Among 245 students (38.5%) who presented with a cough during the outbreak period, the most common cough duration was 2 weeks or more, followed by duration of 1–2 weeks. The most common characteristic was paroxysmal cough, followed by posttussive vomiting. On the basis of the reported clinical symptoms, 84 and 161 students were classified into the probable case (mean age 20.4, range: 18–34 years) and suspected case (mean age 20.0, range: 18–30 years) groups, respectively. The number of cases was greatest in first grade students in the Department of Medicine. Of 245 students with continuous cough, 121 visited a medical institution; of these, 56 were diagnosed with pertussis by physicians. Patients with probable cases were more likely to seek treatment at a medical institution and be diagnosed with pertussis than those with suspected cases. Of the students diagnosed with pertussis, 21 had visited the infection control department at the university hospital. Pertussis DNA was detected in throat swabs obtained from 3 of these students by loop-mediated isothermal amplification, leading to a definitive laboratory diagnosis of pertussis. Most students (534 of 636) could not remember their vaccination status.

**Table 1:** **Characteristics of 636 survey subjects according to clinical diagnosis**

**Characteristics**		**Total (n = 636)**		**No symptoms (n = 391)**		**Suspected cases (n = 161)**		**Probable cases (n = 84)**		P-value*
		**n**	**(%)**	**n**	**(%)**	**n**	**(%)**	**n**	**(%)**	
Department	Medicine	389	61.2	224	57.3	107	66.5	58	69.0	0.037
	Nursing	247	38.8	167	42.7	54	33.5	26	31.0	
Grade	1	164	25.8	85	21.7	54	33.5	25	29.8	0.079
	2	158	24.8	96	24.6	59	36.6	23	27.4	
	3	156	24.5	105	26.9	35	21.7	16	19.0	
	4	158	24.8	105	26.9	33	20.5	20	23.8	
Sex	Male	243	38.2	152	38.9	56	34.8	35	41.7	0.517
	Female	392	61.6	238	60.9	105	65.2	49	58.3	
	Unknown	1	0.2	1	0.3	0	0.0	0	0.0	
Continuous cough	Yes	245	38.5	0		161	100.0	84	100.0	
	Less than 1 week	38	6.0	-		38	23.6	0	0.0	<0.001
	1 week or more and less than 2 weeks	102	16.0	-		102	63.4	0	0.0	
	2 weeks or more	105	16.5	-		21	13.0	84	100.0	
Characters of continuous cough (multiple answers)	Proxysms of coughing	233	36.6	-		152	94.4	81	96.4	0.48
	Inspiratory whooping	22	3.5	-		12	7.5	10	11.9	0.247
	Posttussive vomiting	70	11.0	-		31	19.3	39	46.4	<0.0001
Medical institution	Visited	121	19.0	-		67	41.6	54	64.3	0.0008
	Diagnosed with pertussis	56	8.8	-		30	18.6	26	31.0	0.0323
Self-report DTaP vaccination status										
	No	7	1.1	5	1.3	2	1.2	0	0.0	<0.001
	1 shot	19	3.0	9	2.3	4	2.5	6	7.1	
	2 doses	19	3.0	8	2.0	3	1.9	8	9.5	
	3 doses	10	1.6	5	1.3	5	3.1	0	0.0	
	4 doses	47	7.4	20	5.1	15	9.3	12	14.3	
	Uncertain	534	84.0	344	88.0	132	82.0	58	69.0	

Clinical criteria: (1) cough illness lasting ≧ 2 weeks; (2) paroxysms of coughing; (3) inspiratory “whoop”; (4) post-tussive vomiting.

Suspected case: patient with at least 1 clinical criterion.

Probable case: patient with cough illness lasting ≥ 2 weeks with at least 1 other clinical criterion.

*Chi-square test.

**Figure 1:**
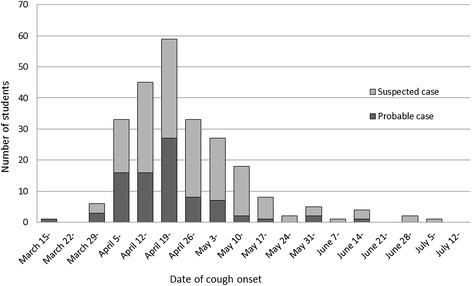
**Epidemic curve based on date of cough onset.** Dark gray and light gray bars indicate suspected and probable cases, respectively.

The epidemic curve based on the date of cough onset is shown in Figure 1. The number of individuals with cough symptoms increased rapidly from early April and decreased after peaking from April 19 to 25. No prophylaxis antibiotics were administered during this time.

**Figure 2:**
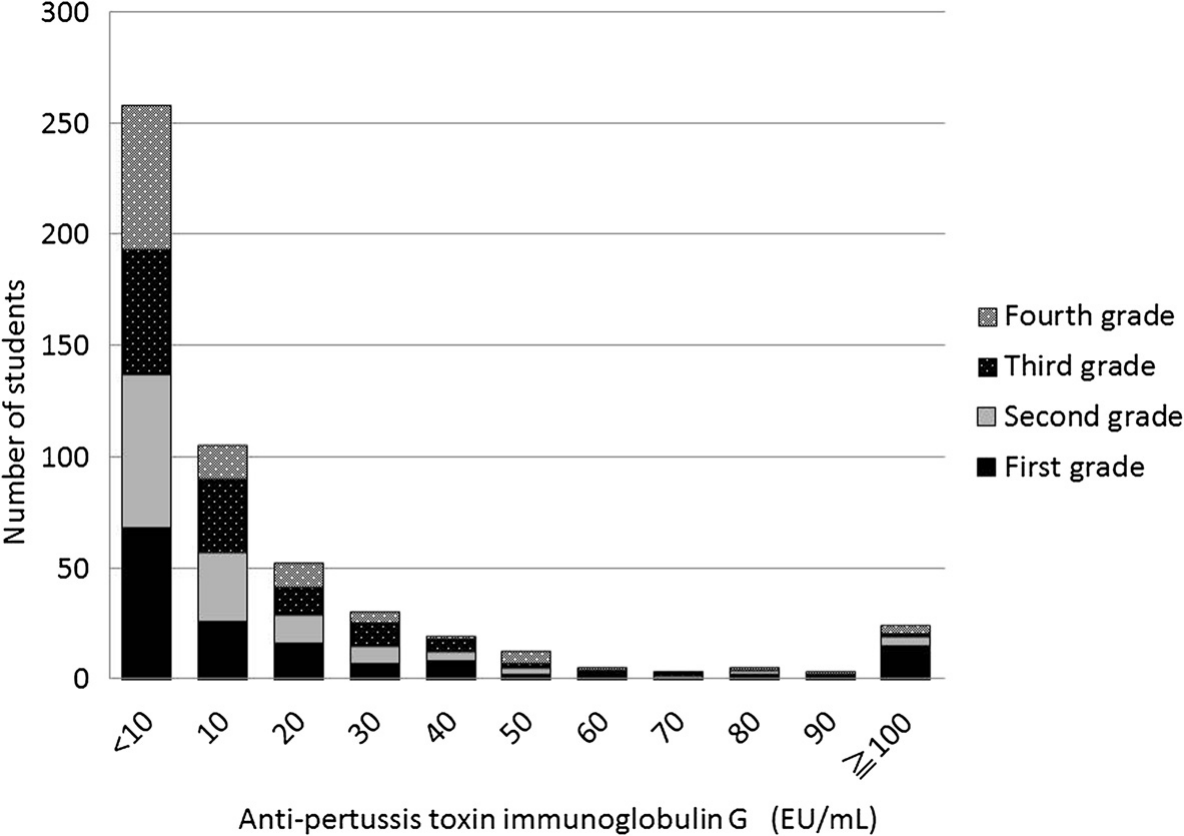
**Distribution of serum pertussis toxin antibodies in 516 students after the outbreak, according to grade.** Black, gray, black with dots, and gray with dots bars indicate first, second, third, and fourth grades, respectively.

Figure 2 shows the distribution of anti-PT antibody titers in 516 students from whom serum was collected after the outbreak, according to grade. Among them 24 subjects’ anti-PT antibody levels were greater than 100 (EU/mL), and the percentage of them was highest in first grade students.

### Evaluation of vaccine effectiveness

Among entire population, 248 first and second grade students whose infant vaccination records could be confirmed by maternity record books were examined according to clinical diagnosis (Table 2). Probable cases were more common in the Department of Medicine. No significant associations were found between grade, sex, and underlying disease and incidence of pertussis. The percentage of students diagnosed with pertussis who had also received the full recommended DTaP vaccination course in infancy was notably low (12.5%). The AR of probable cases per vaccination status was 33% in unvaccinated students and 13.8% in students who had received all 4 doses, indicating that ARs were lowest in students who had received the recommended number of vaccine doses.

**Table 2:** **Comparison of underlying disease and DTaP vaccination according to clinical diagnosis in 248 students with confirmed vaccination records**

**Characteristics**		**Total**	**No symptom (n = 133)**		**Suspected case (n = 77)**		**Probable case (n = 38)**		**P-value** ^*^
		**(n = 248)**	**n**	**(%)**	**n**	**(%)**	**n**	**(%)**	
Department	Medicine	156	76	57.1	51	66.2	29	76.3	0.0075
	Nursing	92	57	42.9	26	33.8	9	23.7	
Grade	1	133	64	48.1	47	61.0	22	57.9	0.1652
	2	115	69	51.9	30	39.0	16	42.1	
Sex	Male	102	53	39.8	30	39.0	19	50.0	0.4784
	Female	146	80	60.2	47	61.0	19	50.0	
History	Allergic rhinitis	59	35	26.3	18	23.4	6	15.8	0.4032
	Anemia	30	15	11.3	9	11.7	6	15.8	0.7474
	Food Allergy	12	7	5.3	3	3.9	2	5.3	0.8979
	Heart disease	2	2	1.5	0	0.0	0	0.0	0.4182
	Liver disease	1	1	0.8	0	0.0	0	0.0	0.6479
	Renal disease	4	4	3.0	0	0.0	0	0.0	0.1724
	Diabetes	1	1	0.8	0	0.0	0	0.0	0.6479
	None	90	48	36.1	32	41.6	10	26.3	0.2778
Vaccination record for DTaP vaccine									
	No	3	1	0.8	1	1.3	1	2.6	0.0742
	1 shot	4	2	1.5	2	2.6	0	0.0	
	2 doses	2	0	0.0	0	0.0	2	5.3	
	3 doses	14	5	3.8	5	6.5	4	10.5	
	4 doses	225	125	94.0	69	89.6	31	81.6	

DTaP: diphtheria, tetanus, and pertussis.

*Chi-square test or Fisher’s exact test.

There were no statistically significant differences in the department, grade, sex, or underlying diseases compared to the completeness of the infant vaccination series. A significantly higher proportion of individuals who did not receive 4 doses of DTaP reported coughing paroxysms. While the clinical characteristics of cough varied, the proportion of students with anti-PT antibody levels greater than 100 EU/mL after the outbreak were similar between those who did and those who did not receive a full vaccine dose (see Additional file 1: Table S1). We examined the RR of the DTaP vaccine for those who had received the government-recommended number of vaccinations in infancy (Table 3). When outcome was defined as probable cases based on the clinical criteria, the RR for students with 4 doses compared to those with fewer than 4 doses or no doses was 0.48 (95% CI: 0.24–0.97); after adjusting for the impact of department the effectiveness was estimated to be 52% (95% CI: 3–76). Similarly, when outcome was defined as meeting at least 1 of the 4 clinical criteria in both probable and suspected cases, the adjusted RR was 0.70 (95% CI: 0.51–0.98). When outcomes were defined as serological diagnosis of pertussis after the outbreak (anti-PT antibody levels greater than 100 EU/mL) or diagnosed at medical institutions, the RRs were 0.64 (95% CI: 0.16–2.52) and 0.74 (95% CI: 0.21–2.61), respectively; no statistically significant protective effect of complete vaccination were detected using these outcome definitions.

**Table 3:** **Relative risks of history of DTaP vaccination for pertussis according to case definition**

**Definition of pertussis**	**Number**	**Case**	**Attack rate (%)**	**Relative risk**	**(95% CI)**	**Relative risk** ^**a**^	**(95% CI)**
Probable cases							
Less than 4 doses or no doses	23	7	30.4	1		1	
4 doses	225	31	13.8	0.45	(0.23-0.91)	0.48	(0.24-0.97)
Probable + Suspected cases							
Less than 4 doses or no doses	23	15	65.2	1		1	
4 doses	225	100	44.4	0.68	(0.49-0.95)	0.70	(0.51-0.98)
Anti- PT antibody titers after outbreak ≥ 100EU/mL							
Less than 4 doses or no doses	23	2	8.7	1		1	
4 doses	225	13	5.8	0.64	(0.16-2.76)	0.64	(0.16-252)
Diagnosed as pertusis at medical institutions							
Less than 4 doses or no doses	23	2	8.7	1		1	
4 doses	225	17	7.6	0.87	(0.21-3.53)	0.74	(0.21-2.61)

DTaP: diptheria, tetanus, and pertussis; CI: confidence interval; PT: pertussis toxin.

Clinical criteria: (1) cough illness lasting ≧ 2 weeks; (2) paroxysms of coughing; (3) inspiratory “whoop”; (4) post-tussive vomiting.

Probable case: a patient who met cough illness lasting ≧ 2 weeks with at least 1 item in the other clinical criteria.

Suspected case: a patient who met at least 1 item in the above 4 clinical criteria.

^a^Adjusted by department using the Mantel-Haenszel method.

## Discussion

The outbreak in this study occurred mainly in first and second grade students on the university campus, with peak incidence in April. Welcoming parties for new pupils or invitations to club activities before and after entrance ceremonies likely contributed to the spread of infection. The outbreak ended without administration of preventive antibiotics. Measures such as self-restraint of club activities, meetings, and ball game tournaments, termination of practical trainings, and active intervention by the health administration center to encourage examination at medical institutions appeared to effectively limit the outbreak. In addition, approximately 1 week of university holidays owing to consecutive holidays in May might also have reduced the spread of infection.

The vaccine effectiveness was 52% for probable cases meeting the clinical criteria for pertussis when students with fewer than 4 or no shots was defined as the reference. It is difficult to directly compare these results with other studies because booster vaccination recommendations vary by country [1,7,8,18], studies use different case definitions [1,18,19], vaccine effectiveness decreases with age-associated decreases in vaccine-induced antibodies [5,20,21], and study subject characteristics may also differ considerably between studies. We report a vaccine effectiveness lower than the 96% effectiveness reported by case–control studies of children in Japan with 3 or more vaccine doses compared to unvaccinated children [15], and about 80% reported by a meta-analysis study of children who received 4 vaccine doses [18]. Considering that the mean age in our study population was 20.4 years, the length of time since the last vaccination may contribute the lower vaccine effectiveness. This observation suggests that replacing the conventional diphtheria and tetanus toxin vaccine administered in adolescence to DTaP might be necessary in Japan. In addition, complete vaccination in infancy is essential, since incomplete vaccination did not show protective effects against pertussis in this study.

In other countries, the DTaP vaccine is administered in early childhood, and a Tdap booster vaccination is administered after early adolescence. Therefore, there are limited reports on the effectiveness of the DTaP vaccine in adolescents and adults. In a case–control study performed during an outbreak at a military school in France, the vaccine effectiveness rates among bio-logically confirmed cases where 5 and 4 DTaP vaccinations were administered was 32% and 22%, respectively [20]. This study also found that effectiveness decreased as the period from the last vaccination increased. On the other hand, 2 case–control studies in adolescents and adults after Tdap booster vaccination reported an effectiveness around 60%; these studies defined patients diagnosed with pertussis by PCR as cases and patients with pertussis-like symptoms but negative by PCR as controls [22,23]. In our study, the effectiveness of the DTaP vaccine was higher than that in a previous report from a US military school and slightly lower than that of Tdap effectiveness. However, because the vaccination series is completed by 2 years of age in Japan, 16 years or more had passed since the last vaccination. We also defined cases based on clinical criteria. Other reasons for these disparate results may be due to the effects of boosters administered during a pertussis outbreak in Japan in 2008 and 2009 [9-13]. Other reasons may include a higher rate of completed vaccine courses: in our study population, the vaccination coverage, or the percentage of the study population that had received 4 vaccine doses, was 94%. Differences in vaccine components [24,25] and vaccination methods (subcutaneous injection in Japan vs. intramuscular injection in the US) may also have contributed to differences in reported results.

Generally, the more precisely defined the outcome, the higher the diagnosis sensitivity [19], and detected effectiveness. In our study, the vaccine effectiveness against probable cases was higher than against suspected cases. However, differences in vaccine effectiveness were not detected by serologically or medically diagnosed cases. In our study, patients with serum anti-PT antibody titers greater than 100 EU/mL at the end of July were considered positive for pertussis, although we could not perform examinations with paired sera to compare levels during the acute phase to the recovery phase of pertussis. Anti-PT antibodies have been reported to decrease particularly rapidly [26], so a patient with pertussis might not show as positive if antibody levels had fallen below this threshold. If many subjects with pertussis were not detected by serological testing, misclassification might occur. Medically diagnosed cases might be confounded by health-related behavior. Not all probable cases visited medical institutions; thus, outcomes were likely biased.

This study had several limitations. First, pertussis was diagnosed only on the basis of clinical criteria. The clinical definition of probable case includes a continuous cough for 14 days or more. In this study, promotion of active interventions and medical examinations at medical institutions occurred during the early phase of the outbreak for the purpose of infection control; as a result, the average cough duration decreased, leading to potential misclassification. Second, we could confirm vaccination records for only half of the study participants. Although misclassification of the vaccination category could be avoided by including only those participants whose maternity record books could confirm vaccination status, the sample size would be quite small. However, since the outbreak of pertussis occurred mainly in first and second grade students in our study, we believe that statistically significant differences in vaccine efficacy could be detected. Third, the past history of pertussis in the study participants is unknown. Since outbreaks of pertussis in high school students have also been recently reported in Japan [12], some students may have been infected with pertussis before entering the university. It is generally believed that a history of pertussis is protective against future pertussis owing to antibodies acquired by natural infection. Inclusion of subjects with a history of pertussis in the group that had not received 4 vaccinations in infancy could lead to underestimated vaccine effectiveness. Finally, hygiene behaviors might confound the association between the vaccination record and onset. For example, if those who did not receive recommended infant vaccinations were also not taught appropriate hygiene behaviors, they might not take prophylactic actions against infection, such as washing hands, wearing facemasks, and avoiding crowds during the pertussis season. However, if these differences exist, the effects are minimal, because vaccine effectiveness was not detected when we examined the association between measles vaccination with pertussis in the subjects of this study (Additional file 2: Table S2).

## Conclusions

An outbreak of pertussis was observed in a population in which the majority of individuals had completed the DTaP vaccine course as infants. The AR was higher in students who did not complete the full infant DTaP vaccine course. The vaccine effectiveness was an estimated 52%, lower than that described in previous reports of children, mostly likely because of decreasing antibody levels in the long period of time since their last DTaP dose. These results suggest the necessity for booster vaccination for teenagers and development of highly efficacious pertussis vaccines.

## Additional files

Additional file 1: Table S1. Characteristics of 248 students with Confirmed DTaP Vaccination History according to Completeness of Infant Vaccination. Additional file 2: Table S2. Relative Risks for Pertussis by History of Measles Vaccination According to Case Definition in 248 Students with Confirmed Vaccination Records.
